# Three-dimensional dentoalveolar characteristics of a labially impacted dilacerated maxillary central incisor using cone-beam computed tomography

**DOI:** 10.1038/s41598-025-10043-9

**Published:** 2025-07-09

**Authors:** Enas Senan Alyafrusee, Bowen Zheng, Abeer A. Almashraqi, Bushra Sufyan Almaqrami, Xiaofeng Yang, Hao Xu, Maged S. Alhammadi, Yi Liu

**Affiliations:** 1 Department of Orthodontics, School and Hospital of Stomatology, Liaoning Provincial Key Laboratory of Oral Disease, China Medical University, Shenyang, 110002 China; 2 Shenyang Clinical Medical Research Center of Orthodontic Disease, Shenyang, 110002 China; 3 Department of Orthodontics and Dentofacial Orthopedics, Faculty of Dentistry, Ibb University, Ibb, Yemen; 4 Department of Clinical Oral Health Sciences, College of Dental Medicine, QU Health, Qatar University, Doha, Qatar; 5 Department of Orthodontics, Ningbo Dental Hospital, Ningbo, 315032 Zhejiang China; 6 Orthodontics Division, Faculty of Medicine and Health Sciences, Taiz University, Taiz, Yemen; 7 Orthodontics and Dentofacial Orthopedics, Department of Preventive Dental Sciences, College of Dentistry, Jazan University, Jazan, Saudi Arabia; 8 Department of Orthodontics, School and Hospital of Stomatology, China Medical University, No. 117, Nanjing North Street, Shenyang, 110002 Liaoning China

**Keywords:** Impacted maxillary central incisor, Dilaceration, Unilateral impaction, Dentoalveolar dimensions, Cone beam computed tomography (CBCT), Anatomy, Medical research

## Abstract

This study aimed to three-dimensionally assess the dentoalveolar characteristics of patients with an impacted dilacerated maxillary central incisor compared to the unaffected side. This cross-sectional study used 35 cone-beam computed tomography scans. Dentoalveolar variables included anterior alveolar ridge height of incisors, alveolar bone thickness, and density, lateral incisors inclination relative to the mid-sagittal plane (MSP), palatal plane (PP), and Frankfort horizontal plane (FHP) with corresponding distances, maxillary arch perimeter, and canine median raphe width. Anterior alveolar bone height was significantly reduced on the impacted side. Additionally, compared with the non-impacted side, there was a significant reduction in the labial alveolar bone thickness of impacted central and ipsilateral lateral incisors. Conversely, there was a considerable increase in the palatal alveolar bone thickness of the impacted central incisor. The labial and palatal alveolar bone density of the impacted central incisors significantly increased compared to that of the non-impacted side. The lateral incisor inclination relative to the PP and FHP and the corresponding distances were reduced considerably on the impacted side, except for lateral incisors inclination to MSP, where it showed a statistically significant greater inclination. The anterior segment of the arch perimeter and the canine median raphe width were also significantly reduced on the impacted side. Significant differences in most dentoalveolar dimensions were observed between the impacted and non-impacted sides in labially impacted maxillary central incisor cases. This study highlights significant dentoalveolar changes in patients with impacted dilacerated maxillary central incisors, underscoring the need for tailored orthodontic treatment.

**Supplementary Information:**

The online version contains supplementary material available at 10.1038/s41598-025-10043-9.

## Introduction

Typically, permanent teeth naturally emerge in the mouth at designated times. Yet, various factors can occasionally complicate this eruption process, causing the tooth to become embedded within the alveolar bone. Among all teeth, the maxillary central incisors are particularly significant due to their crucial role in facial aesthetics, pronunciation, chewing, and psychological well-being^1,2^. Tooth eruption, the axial movement of a tooth from its non-functional position within the alveolar bone to its functional occlusion, is a physiological process critical for the normal development of the alveolar bone. Conversely, tooth impaction can impede the regional development of the alveolar bone^3,4^.

Pathologically, an impacted tooth is defined as an abnormally positioned tooth, either entirely or partially enveloped by mucoperiosteum and bone, and does not erupt in the oral cavity at the expected time and place^5,6^. The teeth most commonly impacted are the mandibular third molars, trailed by the maxillary canines and central incisors. The reported prevalence of impacted maxillary central incisors ranges from 0.06 to 0.20%^2,7^. Impaction is traditionally suspected when a tooth fails to emerge into the dental arch beyond the customary eruption age^8,9^ or if its counterpart has erupted for at least 6 months with a fully developed root^9–12^.

Research studies frequently lack a detailed characterization of alveolar bone dimensions and the mechanical environment at the impaction site. The prevailing hypothesis is that impaction may decrease the masticatory stimulation of the bone. The advent of cone-beam computed tomography (CBCT), which offers high-resolution three-dimensional (3D) views of teeth and bone, now allows us to secure detailed information about alveolar bone dimensions at the site of impaction^13–15^. Forced orthodontic eruption of teeth has been demonstrated to result in adequate development of alveolar bone height^16,17^. On the other hand, other treatment methods, such as guided bone regeneration, have shown less predictability^18,19^.

Modern two-dimensional (2D) radiographs, like periapical, occlusal, and panoramic radiographs, are still the most frequently used methods for initial diagnosis, treatment planning, and localizing unerupted teeth despite newer diagnostic imaging techniques^20^. However, 2D radiographs fall short of accurately determining the exact location of these teeth, their effect on the neighboring teeth, and other adjacent structures, which are necessary for effective treatment planning^21^. Computed tomography (CT) mitigates image superimposition and facilitates the reconstruction of scanned structures in multiple planes, it also enables 3D reconstructions^22,23^. CBCT, a more current, widely accessible modality in dentistry, produces high-grade 3D diagnostic images with minimal distortion. Notably, CBCT provides these advantages at a comparatively low cost and with a significantly lower radiation dose than other CT modalities^24^.

Recent studies have underscored the role of CBCT in understanding the spatial and structural changes associated with impacted anterior teeth, especially in pediatric populations. For example, Pauwels et al.^25^ have discussed the technical limitations of CBCT-derived bone density values, while Mockutė et al.^26^ conducted a systematic review of the morphology of impacted central incisors. While previous research has focused on individual parameters such as root morphology, angulation, or alveolar bone height, there remains a lack of studies comparing dentoalveolar characteristics bilaterally within the same patient. Our study addresses this gap by providing a comprehensive 3D comparison between the impacted and non-impacted sides. Furthermore, recent work by Sharhan et al.^27^ and Chaushu et al.^7^ highlights the importance of bone quality and density in diagnosis and prognosis of impacted teeth. The present study builds on this understanding by evaluating alveolar bone density both labially and palatally, which has not been previously explored in the context of dilacerated central incisor impactions.

This research aimed to investigate the impact of dilacerated maxillary central incisors on various dentoalveolar variables. These variables include anterior alveolar bone height, the inclination and distance of the maxillary lateral incisors relative to reference planes, alveolar bone thickness and density, maxillary arch perimeter, and canine median raphe width on both the impacted and non-impacted sides. The null hypothesis is that there is no significant differences between these variables when comparing the impacted sides to the non-impacted maxillary central incisors. This is the first study to evaluate these dentoalveolar variables on both the impacted and non-impacted sides within the same patients with unilaterally impacted dilacerated maxillary central incisors.

## Materials and methods

### Study design and participants

This cross-sectional study examined CBCT scans of patients from the Department of Orthodontics at China Medical University Hospital, China. This examination followed approval from the Ethics Committee of China Medical University (Approval No. K-2024-017), China. The sample included CBCT scans of subjects with a unilateral impacted dilacerated maxillary central incisors.

Inclusion criteria were as follows: (1) both sexes aged between 8 and 10 years old; (2) the presence of a unilaterally labial impacted dilacerated maxillary central incisor; (3) an impacted central incisor with an apical deviation (dilaceration) is equal or greater than 20° relative to the tooth crown axis, (4) delayed eruption of at least 6 months compared with the contralateral incisor; (5) a fully erupted contralateral incisor with root formation; and (6) scans providing clear image definition.

The exclusion criteria comprised of the following: (1) previous orthodontic treatment; (2) impaction of both maxillary central incisors; (3) dental agenesis; (4) maxillary lesions, trauma, or tumors; (5) cleft palate or lip, and craniofacial abnormalities; (6) the presence of hyper- or hypodontia; and (7) patients with reported systemic bone disease.

### Study sample size

Using G*Power software (V3.1.3; Franz Faul, Universität Kiel, Germany), the sample size calculation was based on a previous study^28^. This study reported the mean inclination of the long axis of the maxillary lateral incisor relative to the Frankfort horizontal plane (FHP) as 62.68 ± 7.88° on the impacted side and 65.31 ± 7.22° on the non-impacted side. To achieve a power level of 95% with a significance level of 5% (α = 0.05), a minimum sample size of 30 participants was necessary. However, the sample size was subsequently increased to 35 patients.

### Three-dimensional imaging

Three-dimensional images were captured using a CBCT (I-CAT^®^; Imaging System, KaVo Company, Germany) following standard protocols by a trained radiographer. The imaging settings included 120 kV and 5 mA with a 23 cm × 17 cm field of view, an exposure time of 17.8 s, a voxel size of 0.3 mm, and a slice thickness of 2 mm. Patients were positioned upright with their teeth in full occlusion during the scan. The scan was conducted on the mid-sagittal plane (MSP), perpendicular to the floor and parallel to the FHP, using a laser guide for alignment. Patients were also instructed to refrain from swallowing during the imaging process.

### Outcomes assessment

**Fig. 1:**
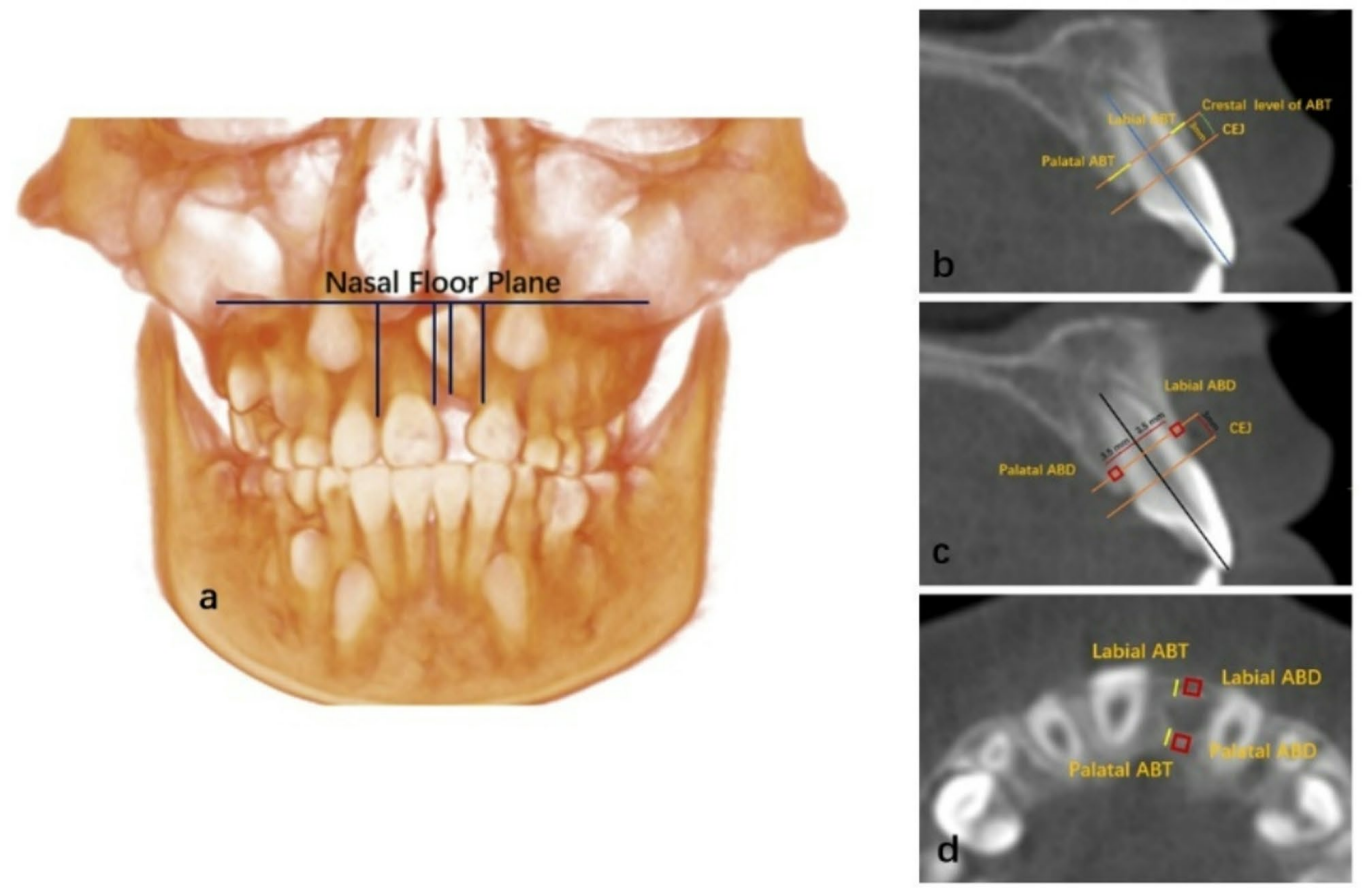
Anterior alveolar bone measurements: a, anterior alveolar ridge height. b, ABT: alveolar bone thickness. c, ABD: alveolar bone density. d, alveolar bone thickness and density of impacted dilacerated maxillary central incisor.

**Fig. 2:**
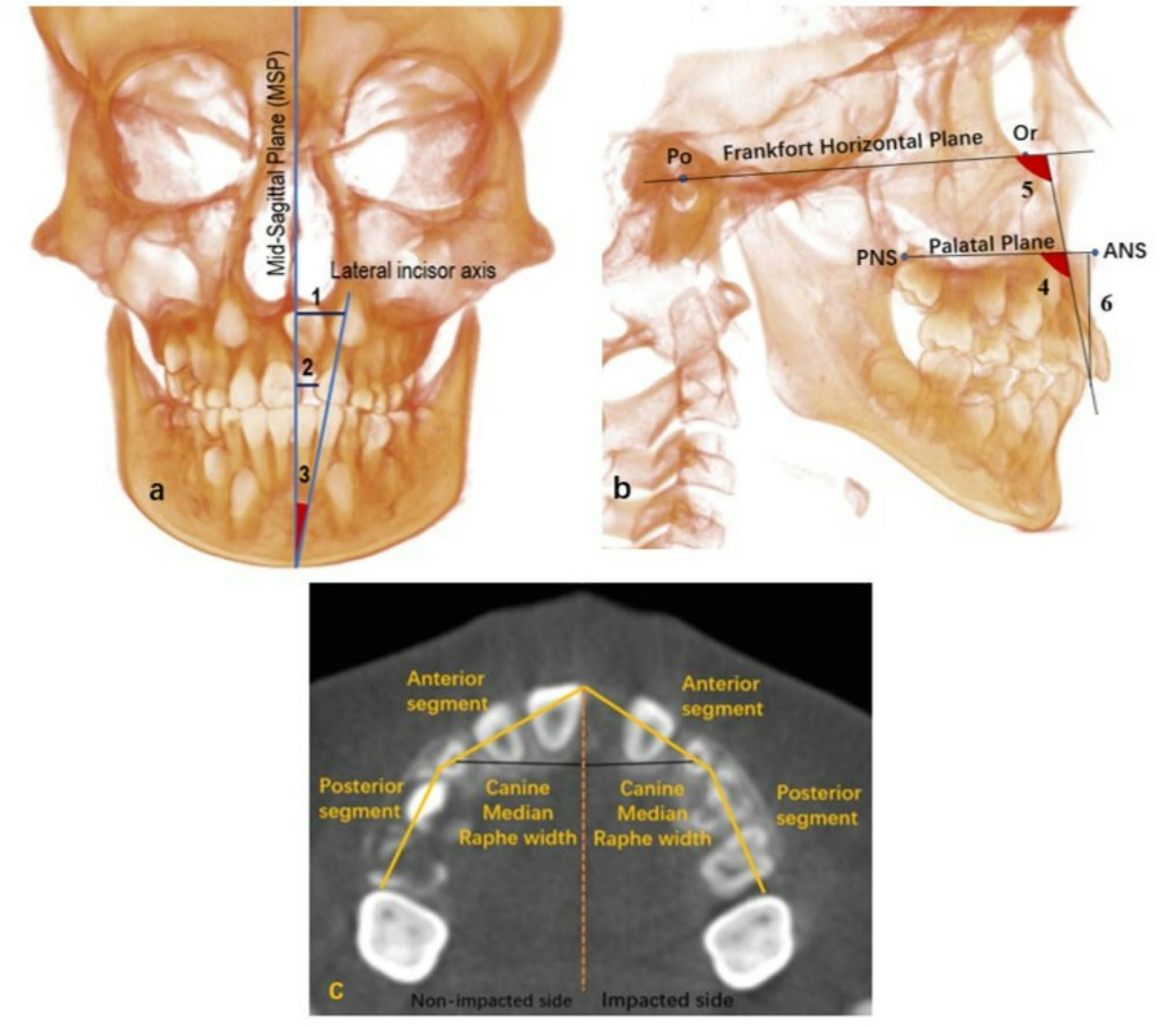
Maxillary lateral incisor and dental arch measurements. a,1. lateral incisor apex-MSP distance, 2. lateral incisor-MSP distance, 3. lateral incisor/MSP angle. b, 4. lateral incisor/PP angle, 5. lateral incisor/FH plane angle, and 6. Lateral incisor-PP distance. c, Maxillary dental arch measurements; anterior and posterior segments of dental arch, and canine median raphe width in impacted and non-impacted sides.

Supplementary Table 1 presents landmarks and reference planes used in this study. The outcomes were divided into: (1) Measurements of the anterior alveolar ridge height for the maxillary incisor teeth, including the normal central incisor and the lateral incisors on both the impacted and non-impacted sides, as shown in Fig. 1a. (2) Alveolar bone thickness and density: axial, coronal, and sagittal planes of the CBCT volume were reoriented to be perpendicular to the long axis of each tooth under assessment. Three-dimensionally, a reference line was drawn from the sagittal view, connecting the labial and palatal cementoenamel junction (CEJ). Labial and palatal alveolar bone thickness (ABT) around the incisor teeth (contralateral central incisor (CCI), lateral incisors of both impacted and non-impacted sides) was measured at one level with distances of 3 mm from the CEJ in the apical direction as illustrated in Fig. 1b. The alveolar bone density (ABD) was measured at a distance of 3 mm from the CEJ in the apical direction and 3.5 mm from the midline (tooth axis) labially and palatally, as seen in the sagittal view Fig. 1c. This section was referred to as the crestal section. The ABT was measured in mm, and the alveolar bone density value was registered in a spot diameter of 1 mm^2^ each labially and palatally. This size of the measured area was adjusted to equal in all cases. These measurements were taken using sagittal slices, where the maxillary incisor teeth have the maximum labio-palatal width. Multiplanar reconstruction methods were employed to help identify landmarks in the sagittal slices. For the impacted central incisor, in the axial view, the ABT was measured at the maximum cortical bone width area labially and palatally, and the bone density was registered in a spot diameter of 1 mm^2^ each labially and palatally, as shown in Fig. 1d. (3) The maxillary lateral incisors inclination relative to the MSP, PP, and FHP, as well as the corresponding distances related to MSP and PP, as illustrated in Fig. 2a and b and described in Supplementary Table 2. The maxillary dental arch measurements are illustrated in Fig. 2c and described in Supplementary Table 2. The angle between the crown axis of the impacted maxillary central incisor and the tooth axis of the contralateral tooth indicated the direction of the long axis of the impacted tooth crown. In contrast, the angle between the crown and root axis of the impacted maxillary central incisor indicated the degree of root axis curvature. All measurements were conducted under the supervision and guidance of oral and maxillofacial radiologists with more than 15 year of experience. To ensure the measurements’ reliability, intra-observer, and inter-observer reliability were assessed randomly after 3 weeks using the records of ten patients.

### Statistical analysis

The data were analyzed using SPSS version 26.0. Descriptive statistics, including the mean and standard deviation, were calculated for each variable in both groups. The Shapiro-Wilk test was used to determine whether the data followed a normal distribution. Since some data were normally distributed while others were not, the means were compared using either a paired t-test or the Wilcoxon Signed-Rank Test. The Spearman Rank Correlation test was used to assess the correlation between the direction of impacted teeth and root axis curvature with measurements of both impacted and non-impacted sides. Significance was set at *P* < 0.05. The reliability between repeat observations was assessed using Cronbach’s Alpha (intra-class correlation coefficients, ICC). 

## Results

The study comprised a total of 35 patients, including 18 females and 17 males. The results of the inter-examiner reliability analysis can be found in Supplementary Table 3. The ICC displayed high consistency (*P* < 0.001). All ICC values surpassed the suggested cut-off of 0.95, signifying minimal measurement error in this study.

There was statistically significantly less alveolar bone height on the impacted side for both central and lateral incisors (*P* < 0.001), as shown in Table 1. Furthermore, there was notably more labial bone thickness and less palatal bone thickness around the central incisors on the non-impacted side than the impacted ones (*P* < 0.05). The ipsilateral lateral incisor also had significantly less labial bone thickness (*P* < 0.001). There was a statistically significant increase in bone density around the impacted central incisor, both labially and palatally compared to that of the CCI (*P* < 0.05).

**Table 1:** Results of anterior alveolar ridge height, thickness, and density between the impacted and non-impacted sides.

Alveolar bone parameter			Impacted side Group		Non-impacted side Group		Impacted side Group - Non-impacted side Group		*p*
			Mean	SD	Mean	SD	Mean	SD	
Anterior alveolar ridge height of central incisor (AARHCI) (mm) †			12.94	1.73	14.62	1.86	-1.67	1.02	0.000***
Anterior alveolar ridge height of lateral incisor (AARHLI) (mm) †			12.80	2.19	13.89	2.00	-1.09	0.84	0.000***
Alveolar bone thickness (ABT) (mm) †	Central incisor	Labial surface	1.02	0.43	1.26	0.41	-0.25	0.57	0.014*
		Palatal surface	2.58	0.76	2.06	0.62	0.52	1.00	0.004**
	Lateral incisor	Labial surface	0.96	0.36	1.61	0.55	-0.65	0.69	0.000***
		Palatal surface	1.71	0.57	1.75	0.68	-0.04	0.68	0.800
Alveolar bone density (ABD) (mm^2^) ††	Central incisor	Labial surface	789.23	129.01	633.37	187.99	155.86	200.20	0.000***
		Palatal surface	805.34	136.11	647.89	258.73	157.46	263.87	0.003**
	Lateral incisor	Labial surface	555.40	225.50	546.63	209.64	8.77	290.46	0.859
		Palatal surface	574.29	197.16	563.14	180.15	11.14	241.61	0.787

SD; standard deviation, †; Paired t-test, ††; Wilcoxon signed-rank test. *P < 0.05; **P < 0.01; ***P < 0.001.

In Table 2, the lateral incisor exhibited a statistically significant greater inclination with MSP, while it had a statistically significant lesser inclination with PP and FHP (*P* < 0.05). The corresponding distances associated with MSP and PP also indicated a statistically significant lesser lateral incisor distance concerning both planes (*P* < 0.05). Yet, the distance from the apex of the lateral incisor to the MSP was statistically greater on the impacted side (*P* < 0.001). Additionally, there was a statistically significant lesser anterior arch segment and canine median raphe on the impacted side (*P* < 0.001). However, the two sides had no significant difference in the posterior arch segment.

In Tables 3 and 4, the Spearman Rank Correlation test was utilized to evaluate the correlation between the direction of impacted teeth and root axis curvature with dentoalveolar dimensions on both impacted and non-impacted sides. The dentoalveolar dimensions on the non-impacted side displayed no significant correlation, with a *P* > 0.05. In contrast, there was a highly significant correlation between the direction of the impacted tooth and root axis curvature with the lateral incisors/MSP angle measurement on the impacted side, characterized by *P* = 0.001 and *P =* 0.003, respectively.

**Table 2:** Results of maxillary lateral incisor measurements and other dental arch parameters between the impacted and non-impacted sides.

Measurement	Impacted side Group		Non-impacted side Group		Impacted side Group - Non-impacted side Group		*p*
	Mean	SD	Mean	SD	Mean	SD	
Maxillary lateral incisor measurements							
Lateral incisor/MSP (°) †	9.71	3.01	6.91	4.18	2.80	-1.17	0.002**
Lateral incisor/PP (°) †	57.80	4.42	65.78	3.40	-7.98	5.23	0.000***
Lateral incisor/FHP (°) ††	58.99	4.80	68.64	3.71	-9.66	4.32	0.000***
Lateral incisor-MSP (mm) †	6.34	2.10	8.19	1.90	-1.85	0.21	0.002**
Lateral incisor apex-MSP (mm) †	14.66	2.15	11.77	0.68	2.89	2.29	0.000***
Lateral incisor-PP (mm) †	18.18	3.04	19.56	3.38	-1.37	3.75	0.037*
**Dental arch parameters**							
Anterior Segment of Arch Perimeter (ASAP) (mm) †	14.39	1.66	16.30	1.50	-1.91	1.93	0.000***
Posterior Segment of Arch Perimeter (PSAP) (mm) †	21.82	1.82	21.83	1.56	-0.02	1.33	0.941
Canine Median Raphe Width (CMRW) (mm) †	13.07	1.53	14.71	1.63	-1.64	1.43	0.000***

SD; standard deviation, †; Paired t-test, ††; Wilcoxon signed-rank test. *P < 0.05; **P < 0.01; ***P < 0.001.

**Table 3:** Correlation between the measurements of impacted maxillary central incisors and impacted side measurements.

Measurements			Direction of the long axis of the crown		Root axis curvature	
			*r*-value	*p*-value	*r*-value	*p*-value
AARHCI (mm)			0.34	0.043	0.24	0.162
AARHLI (mm)			0.30	0.078	0.09	0.626
Alveolar bone thickness (ABT) (mm)	Central incisor	Labial surface	-0.09	0.604	0.07	0.676
		Palatal surface	-0.16	0.347	-0.18	0.296
	Lateral incisor	Labial surface	0.29	0.090	0.13	0.474
		Palatal surface	-0.01	0.966	-0.03	0.888
Alveolar bone density (ABD) (mm^2^)	Central incisor	Labial surface	-0.17	0.320	-0.06	0.748
		Palatal surface	0.05	0.796	0.25	0.149
	Lateral incisor	Labial surface	0.27	0.115	-0.06	0.742
		Palatal surface	-0.02	0.928	0.02	0.928
Lateral Incisors/ MSP (°)			0.53	0.001 ***	0.50	0.003 **
Lateral incisors/ PP (°)			0.12	0.483	0.06	0.720
Lateral incisors/ FHP (°)			0.04	0.837	0.05	0.760
Lateral incisor-MSP (mm)			0.01	0.934	0.03	0.887
Lateral incisor apex-MSP (mm)			-0.15	0.388	-0.16	0.372
Lateral incisor-PP (mm)			0.04	0.783	-0.10	0.563
ASAP (mm)			-0.19	0.274	-0.01	0.942
PSAP (mm)			0.34	0.144	0.18	0.305
CMRW (mm)			0.04	0.806	-0.06	0.728

Spearman Rank Correlation test was used to assess the correlation between the direction of impacted teeth and root axis curvature with measurements of the impacted side. **P < 0.01; ***P < 0.001.

**Table 4:** Correlation between the measurements of impacted maxillary central incisors and the non-impacted side measurements.

			Direction of the long axis of the crown		Root axis curvature	
			*r*-value	*p*-value	*r*-value	*p*-value
AARHCI (mm)			0.18	0.309	0.15	0.392
AARHLI (mm)			0.30	0.081	-0.04	0.828
Alveolar bone thickness (ABT) (mm)	Central incisor	Labial surface	0.09	0.612	0.02	0.904
		Palatal surface	0.30	0.085	0.31	0.072
	Lateral incisor	Labial surface	0.00	0.990	-0.02	0.909
		Palatal surface	-0.06	0.737	-0.00	0.995
Alveolar bone density (ABD) (mm^2^)	Central incisor	Labial surface	-0.01	0.937	-0.02	0.889
		Palatal surface	0.31	0.067	0.12	0.493
	Lateral incisor	Labial surface	-0.18	0.298	-0.11	0.526
		Palatal surface	-0.32	0.057	-0.22	0.209
Lateral Incisors/ MSP (°)			-0.08	0.646	0.14	0.427
Lateral incisors/ PP (°)			0.03	0.879	-0.17	0.333
Lateral incisors/ FHP (°)			-0.35	0.140	-0.03	0.866
Lateral incisor-MSP (mm)			-0.36	0.132	-0.13	0.459
Lateral incisor apex-MSP (mm)			0.14	0.419	-0.06	0.723
Lateral incisor-PP (mm)			-0.01	0.923	-0.30	0.085
ASAP (mm)			0.81	0.297	0.08	0.661
PSAP (mm)			-0.02	0.909	-0.03	0.870
CMRW (mm)			0.17	0.322	-0.02	0.915

Spearman Rank Correlation test was used to assess the correlation between the direction of impacted teeth and root axis curvature with measurements of the non-impacted sides.

## Discussion

This study revealed significant differences in alveolar bone dimensions (height, thickness, and density), the inclination of the maxillary lateral incisor, distances to reference planes, the perimeter of the maxillary dental arch, and the width of the canine median raphe between the impacted and non-impacted sides. The primary objective was to compare these variables within the same patient. These differences are crucial for understanding the mechanical environment on impacted and non-impacted sites.

Our findings are consistent with previous reports on the adverse effects of impaction on alveolar bone height and labial thickness^29,30^. However, to our knowledge, this is the first study that systematically assesses both bone thickness and density on labial and palatal surfaces, offering a complete picture of the osseous environment around the impacted incisor. While Hu et al.^28^ reported insignificant differences in lateral incisor inclination relative to FHP, our study, with a larger sample size, found statistically significant changes. This highlights the importance of sample power and imaging precision, particularly when using CBCT for three-dimensional evaluations. Additionally, our findings on increased bone density on the impacted side contrast with some previous assumptions that reduced density is the primary obstacle to eruption. This suggests that denser bone may hinder eruptive potential, a hypothesis supported by Pauwels et al.^25^ and warrants further biomechanical investigation. Compared to Chaushu et al.^31^, who relied on 2D imaging, our use of CBCT provided higher spatial accuracy and reduced observer variability, enabling clearer identification of inclination differences and distance measurements. This methodological advancement further emphasizes the originality and clinical relevance of our study.

In this study, we compared the dimensions of the dentoalveolar between the impacted and non-impacted sides in subjects with unilateral impacted dilacerated maxillary central incisors. A significant decrease in the anterior alveolar bone height for the central and lateral incisors on the impacted side was observed. This reduction is likely due to decreased osteogenesis and osteoclastogenesis, possibly resulting from a lack of stimulation by the unerupted maxillary central incisor. This diminished bone formation may also affect the hemi-arch width, especially at the canine level^29,30^. Cahill and Pilipili et al.^32–34^ have reported similar observations in their studies, emphasizing the essential role of tooth eruption in the growth of alveolar bone. The functional matrix theory also corroborates this, suggesting that the tooth organ’s presence is vital for normal bone growth in the anterior premaxillary area^35^.

Concerning the thickness and density of the alveolar bone, this study observed a reduction in labial bone thickness for the impacted central and lateral incisors compared to contralateral non-impacted teeth. Conversely, an increase in palatal bone thickness was noted for the impacted central incisor. To our knowledge, this represents the first study to measure and compare labial and palatal bone density and thickness both at the site of impaction (the edentulous space intended for the impacted maxillary central incisor) and on the contralateral side with a normally positioned central incisor. Regarding alveolar bone density, our study demonstrated an increase in labial and palatal bone density around the impacted maxillary central incisor compared to CCI. The difference in bone density between the non-impacted and impacted groups supported the hypothesis that higher bone density prevents the normal growth of the developing tooth, eventually leading to the failure of the eruption process^25,27^. The increase in bone density could indeed reflect sclerosis, which might result from chronic inflammation or mechanical stress. These factors could lead to the hardening of the bone around the impacted tooth as a reaction to prolonged impaction. Chronic inflammation, often caused by an inability of the tooth to erupt, can trigger the activation of osteoblasts, leading to excessive bone formation as part of the body’s attempt to wall off the impacted tooth. Similarly, mechanical stress from the tooth’s attempted eruption, despite being impeded, may stimulate bone remodeling, resulting in increased bone density^36^. In our study, while we observed a statistically significant increase in bone density, the interpretation of this finding remains cautious, as it suggests a complex interaction between biological and mechanical factors rather than a straightforward indication of bone health.

Regarding the clinical implications, clinicians should take anchorage planning and the correct force level into account when performing forced eruption of buccally impacted canines with high bone density to reduce unwanted movements and ensure the best treatment results. Also, it is essential to consider whether this finding should influence treatment planning, particularly regarding the surgical approach. If the increased bone density reflects sclerosis, it could complicate the orthodontic and surgical management of impacted incisors. In such cases, it may be necessary to consider pre-surgical interventions such as osteotomy or bone reduction to facilitate tooth eruption. Additionally, the potential for bone dehiscence or root resorption could be higher in areas with abnormally dense bone, making it essential to assess bone quality before deciding on a treatment approach.

Table 2 presents data on the orientation of the maxillary lateral incisors relative to the MSP, PP, and FHP. The study identified significant variations in the inclination of the maxillary lateral incisors between impacted and non-impacted sides, with the angle notably smaller on the affected side. Specifically, the inclination of the ipsilateral lateral incisor relative to the MSP demonstrated a significantly increased angle compared to the contralateral side. This corresponds with previous research^24^ suggesting that lateral incisors next to an impacted central incisor tend to be more angled towards the MSP than those on the non-impacted side. This can be attributed to the mesial drift of the ipsilateral lateral incisor towards the space of the impacted central incisor, causing the apex to position more distally. As a result, malposition of the canine becomes more prevalent and severe the further the apex displaces distally, in line with earlier findings^26^. While these correlations are statistically significant, the strength (r-values) of the correlations does not necessarily imply a direct causal relationship, as correlations do not always equate to causality. We have emphasized that clinicians should interpret these findings cautiously and that these correlations offer valuable insights but should not be used as sole determinants in treatment planning, except in cases where this angulation is very high, which might cause considerable root resorption of the lateral incisor during treatment and should be monitored closely.

The study also revealed a significant decrease in the inclination of the ipsilateral lateral incisor relative to the PP compared to the non-impacted side. These results correspond with Chaushu’s research^31^, where panoramic films were used to study the inclination of maxillary lateral incisors in patients having impacted dilacerated maxillary central incisors. Chaushu reported a significant decrease in the angle of the ipsilateral lateral incisor to the PP compared to the contralateral side. Considering the inclination relative to the FHP, our findings are compatible with those of Hu et al.^28^, who reported insignificant differences in the inclination of the maxillary lateral incisors on the impacted side compared to the contralateral side. They suggested that this insignificant result was due to a small sample size and advocated for a larger sample slate in future studies. The research revealed a notable decrease in the incisal distance of the ipsilateral lateral incisor to the MSP compared to the contralateral tooth, with an increase in the apical distance. Precisely, the incisal edge of the ipsilateral lateral incisor was 1.85 mm closer to the midline, while its root was displaced distally by an average of 2.29 mm compared to the contralateral lateral incisor. These outcomes corroborate with a previous study^31^, which utilized panoramic films to examine unerupted maxillary permanent canines in patients with impacted central incisors due to factors like obstruction, dilaceration, and trauma. They reported that the dilacerated subgroup had a mesial drift of the ipsilateral lateral incisor toward the midline, with the incisal edge tipping 3 mm closer and the apex drifting distally by an average of 5 mm. However, the study noted that linear measurements on panoramic radiographs could be inconclusive and subject to observer bias. In contrast, the current study employed CBCT for more precise measurements and improved recognition of anatomical landmarks, which is crucial for complicated cases involving impacted teeth.

The current study observed that the ipsilateral lateral incisor showed significant mesial angulation, causing it to migrate toward the midline. This change can directly affect the eruption path of the adjacent canine, leading to a lack of space for the canine, and may result in its malposition, impaction, or delayed eruption. This is consistent with Becker et al.‘s canine guidance theory^37^, which posits that the lateral incisor plays a pivotal role in guiding the eruption of the canine. Thus, a greater distal displacement of the apex of the lateral incisor can interfere with the canine’s normal eruption path, and is associated with increased prevalence and severity of canine malposition, potentially requiring early intervention to prevent canine impaction^31^. In addition to these localized effects, the overall maxillary arch form could be compromised due to the lateral incisor’s movement. As observed in our study, the reduced arch perimeter and altered transverse dimension can exacerbate crowding and lead to transversal discrepancies, especially if the space needed for the canine’s eruption is insufficient.

The study also found a significant reduction in the anterior segment of the arch perimeter on the impacted side compared to the non-impacted side. This reduction could be attributed to the early loss of the primary central incisor on the impacted side and the mesial migration of the posterior teeth, leading to a decreased arch perimeter^38^. Additionally, this study found significant differences in canine to median raphe widths between the impacted and non-impacted sides. The distance from the mid-palatine raphe to the canine cusp tip was significantly lower on the impacted side. This could indicate that tooth impaction may cause various alterations in the surrounding tissues, and can lead to transversal alveolar bone alterations, which can be addressed through appropriate treatment. It is advised that clinicians correct any transverse discrepancies that may be present. The decrease in anterior arch perimeter and canine median raphe width reflects a loss of space in the maxillary arch, a direct consequence of the impacted tooth. This space loss can present significant challenges in orthodontic treatment, particularly in space management for erupting teeth, especially when the canine is involved. These findings have not previously been explored, making this study the first to highlight these aspects and to achieve such results. Clinically, such space loss may lead to crowding, improper tooth alignment, and complicated tooth eruption, potentially requiring more complex orthodontic strategies such as extraction, expansion, or surgical intervention^29,39–41^. The impacted maxillary central incisor can directly impact the position of adjacent teeth by blocking their normal eruption and influencing their spatial arrangement. In contrast, the mesial migration of posterior teeth due to primary tooth loss is a more indirect effect, where the loss of the primary tooth may lead to shifts in the arch, creating space issues for the permanent teeth. While both processes contribute to dentoalveolar changes, they are distinct mechanisms that should be considered separately in treatment planning.

The study found no significant correlation between the orientation of the impacted tooth and the curvature of the root axis concerning the dentoalveolar dimensions on the non-impacted side. This suggests that the impacted tooth does not influence the structure of the dentoalveolar on the unaffected side. However, significant correlations were noted between the affected tooth’s orientation and the curvature of the root axis in context with the lateral incisors/MSP angle. This implies that the impacted central incisor can impact the positioning and angulation of the adjacent lateral incisor, potentially causing functional and aesthetic issues. This observation aligns with past research^26^, suggesting that lateral incisors adjacent to an impacted central incisor often exhibit greater angulation toward the MSP than those on the non-impacted side. The noteworthy effect on the angle of the lateral incisors/MSP underlines the importance of considering the orientation and curvature of the impacted teeth when planning orthodontic or surgical treatments to prevent adverse impacts on neighboring teeth.

The clinical relevance of our findings lies in their potential to inform early diagnosis and individualized treatment planning. The significant differences observed in alveolar bone height, thickness, and density, as well as in lateral incisor inclination and dental arch dimensions, highlight the biomechanical consequences of delayed eruption due to impaction. These parameters are critical when evaluating space availability, eruption path, and risk of complications such as root resorption or unfavorable canine positioning. Clinicians can use this 3D data to guide the timing and method of intervention for instance, considering early interceptive treatment to preserve space or reduce asymmetry. Furthermore, increased alveolar bone density in the impaction region may affect the feasibility and duration of the forced eruption, thus influencing surgical and orthodontic strategies. Our within-patient side-by-side comparisons underscore the importance of individualized assessment, highlighting the limitations of relying solely on the presumed symmetry of the contralateral side.

## Conclusions

The null hypothesis was rejected, the CBCT measurements endorse the conclusions that there is a significant reduction in the anterior alveolar bone height and labial bone thickness on the side affected by impaction; this includes both the impacted central and ipsilateral incisors, while the palatal bone thickness is notably increased in the region of the impacted central incisor. Moreover, the labial and palatal alveolar bone density was significantly higher, particularly in the affected maxillary central incisor region, compared to the side without impaction. The maxillary lateral incisor on the impacted side was found to be more mesially angulated, with its apex positioned more distally. Significantly, the arch perimeter and the width from the canine to the median raphe were reduced on the impacted side. A significant correlation was also identified between the impacted central incisor’s orientation and the lateral incisors’ angulation concerning the MSP.

## Electronic supplementary material

Below is the link to the electronic supplementary material.

Supplementary Material 1

Supplementary Material 2

Supplementary Material 3

## Data Availability

The data utilized and/or analyzed in this study can be obtained from the corresponding author upon reasonable request.
