# Supplementary material for: Three-dimensional dentoalveolar characteristics of a labially impacted dilacerated maxillary central incisor using cone-beam computed tomography
Source: Sci Rep. 2025 Jul 9;15:24669. doi: 10.1038/s41598-025-10043-9 (PMC12241505; doi:10.1038/s41598-025-10043-9)
Supplement: Supplementary file 1 — Supplementary Material 1 [file 41598_2025_10043_MOESM1_ESM.docx]

| **Supplementary Table 1:** The landmarks and reference planes used in this study | | |
| --- | --- | --- |
| **Identification** | **Abbr.** | **Definition** |
| **Landmarks** | | |
| Nasion | N | The center of the anterior and superior frontonasal suture. |
| Sella | S | The center of the hypophyseal fossa in the middle cranial fossa (Sella turcica). |
| Basion | Ba | The posterior tip of the cranial base. Sagittally the most inferior posterior point of the foramen magnum. |
| Orbitale | Or | The left or right lowest point on the inferior border of the orbit. |
| Porion | Po | The right and left most outer and superior bony points of the external auditory meatus. |
| Anterior nasal spine | ANS | The tip of the median sharp bony process of the palatine bone in the hard palate. |
| Posterior nasal spine | PNS | The tip of the posterior spine of the palatine bone of the hard palate. |
| Midpoint of edentulous bony ridge of impacted upper central incisor | U1bp | A midpoint on the edentulous bony ridge of impacted central incisor to the nasal floor reference plane. |
| Incisal edge of upper lateral incisor | ULi | Upper lateral incisor’s incisal edge. |
| Root apex of upper lateral incisor | ULa | Upper lateral incisor’s root apex. |
| Mesial bony point of upper central/lateral incisor | U1/L mp | A point on the mesial bony ridge of incisor tooth. |
| **Reference lines and planes** | | |
| Mid-Sagittal plane | MSP | A plane constructed through Sella and Nasion and perpendicular to the horizontal plane. |
| Palatal plane | PP | Line extending from Anterior nasal pine to Posterior nasal spine. |
| Frankfort Horizontal plane | FHP | A plane deﬁned by three landmarks: right Orbitale and Porion and left Porion. |
| Long axis of the upper lateral incisor | ULax | Line that extends from the incisor edge to the root apex of maxillary lateral incisor. |
