# Supplementary material for: Three-dimensional dentoalveolar characteristics of a labially impacted dilacerated maxillary central incisor using cone-beam computed tomography
Source: Sci Rep. 2025 Jul 9;15:24669. doi: 10.1038/s41598-025-10043-9 (PMC12241505; doi:10.1038/s41598-025-10043-9)
Supplement: Supplementary file 2 — Supplementary Material 2 [file 41598_2025_10043_MOESM2_ESM.docx]

| **Supplementary Table 2:** The measurement used in this study | |
| --- | --- |
| **Measurements** | **Definition** |
| **Alveolar bone height (mm)** | |
| Anterior alveolar ridge height of maxillary central and lateral incisors (AARHCI/ AARHLI) | The distance was obtained three-dimensionally by extending a line from a point on the mesial bony ridge of each tooth to the nasal floor reference plane, parallel to the mid-sagittal plane. For the impacted central incisor, the measurement was taken from a midpoint on the edentulous bony ridge to the nasal floor reference plane. |
| **Bone thickness of maxillary Central/lateral Incisors (mm)** | |
| Labial alveolar bone thickness (ABT) | Measured at a distance of 3 mm from the CEJ in the sagittal view. These sections were referred to as the crestal sections. As for the impacted central incisor, the measurements were performed on the axial slice at which the impacted central incisor has the maximum labial width |
| Palatal alveolar bone thickness (ABT) | Measured at a distance of 3 mm from the CEJ in the sagittal view. These sections were referred to as the crestal sections. As for the impacted central incisor, the measurements were performed on the axial slice at which the impacted central incisor has the maximum palatal width |
| **Bone density of maxillary Central/lateral Incisors (mm^2^)** | |
| Labial alveolar bone density (ABD) | Measured at a distance of 3mm from the CEJ in the apical direction, and a distance of 3.5 mm from the midline (tooth axis) labially. |
| Palatal alveolar bone density (ABD) | Measured at a distance of 3mm from the CEJ in the apical direction, and a distance of 3.5 mm from the midline (tooth axis) palatally. |
| **Maxillary Lateral Incisor measurements** | |
| Lateral Incisors/ MSP angle (°) | Coronally, the inferior angle is formed by the long axis of the lateral incisor and the Palatal plane. |
| Lateral incisors/ pp angle (°) | Sagittally, the inferior angle is formed by the long axis of the lateral incisor and the Palatal plane. |
| Lateral incisors/ FHP (°) | Sagittally, the inferior angle is formed by the long axis of the lateral incisor and the Frankfort Horizontal plane. |
| Lateral incisor/MSP (mm) | The distance was obtained three-dimensionally by extending a line from the most prominent mesioincisal point of the maxillary lateral incisors to the MSP horizontally on the side of the impacted central incisor and the side without impaction. |
| Lateral incisor apex/MSP (mm) | The distance was obtained three-dimensionally by extending a line from the center point of the apical foramen of the maxillary lateral incisors to the MSP horizontally on the side of the impacted central incisor and the side without impaction. |
| Lateral incisor/PP (mm) | The distance was obtained three-dimensionally by extending a line from the incisal edge midpoint of upper lateral incisors parallel to the midsagittal plane till the palatal plane vertically on the side of the impacted central incisor and the side without impaction. |
| **Maxillary dental arch (mm)** | |
| Anterior segment of arch perimeter (ASAP) | A distance extended from the midline of the maxillary arch to the distal point of the canines. |
| Posterior segment of arch perimeter (PSAP) | A distance extended from the distal point of the canines to the mesial point of the first molars |
| Canine to median raphe (CMRW) | Distance in millimeters from the middle palatine raphe to the canine cusp tip on each side, measured in the axial view. |
