# Supplementary material for: Three-dimensional dentoalveolar characteristics of a labially impacted dilacerated maxillary central incisor using cone-beam computed tomography
Source: Sci Rep. 2025 Jul 9;15:24669. doi: 10.1038/s41598-025-10043-9 (PMC12241505; doi:10.1038/s41598-025-10043-9)
Supplement: Supplementary file 3 — Supplementary Material 3 [file 41598_2025_10043_MOESM3_ESM.docx]

| **Supplementary Table** **3:** Reliability analysis of the three-dimensional measurements used in the study | | | | | | | | |
| --- | --- | --- | --- | --- | --- | --- | --- | --- |
| **Measurements** | **Intra-observer error** | | | | **Inter-observer error** | | | |
|  | ICC | TEM | rTEM | R | ICC | TEM | rTEM | R |
| AARH (mm) | 0.981 | 0.31 | 7.13 | 0.987 | 0.989 | 0.22 | 5.18 | 0.992 |
| Lateral incisor/PP (°) | 0.989 | 0.52 | 2.46 | 0.992 | 0.987 | 0.52 | 2.45 | 0.991 |
| Lateral incisor/MSP (°) | 0.958 | 0.56 | 16.06 | 0.971 | 0.995 | 0.19 | 5.61 | 0.997 |
| Lateral incisor/FHP (°) | 0.983 | 0.61 | 2.86 | 0.988 | 0.990 | 0.45 | 2.11 | 0.993 |
| Lateral incisor-MSP (mm) | 0.970 | 0.30 | 12.99 | 0.979 | 0.968 | 0.34 | 14.32 | 0.977 |
| Lateral incisor apex-MSP (mm) | 0.982 | 0.22 | 4.06 | 0.988 | 0.976 | 0.26 | 4.84 | 0.983 |
| Lateral incisor-PP (mm) | 0.994 | 0.22 | 3.56 | 0.995 | 0.996 | 0.17 | 2.72 | 0.997 |
| ABT (mm) | 0.960 | 0.10 | 24.90 | 0.971 | 0.984 | 0.05 | 14.40 | 0.989 |
| ABD (mm^2^) | 0.981 | 17.10 | 6.64 | 0.987 | 0.996 | 7.79 | 3.05 | 0.997 |
| ASAP (mm) | 0.971 | 0.15 | 3.46 | 0.980 | 0.966 | 0.15 | 3.61 | 0.976 |
| PSAP (mm) | 0.995 | 0.11 | 1.54 | 0.996 | 0.987 | 0.18 | 2.43 | 0.991 |
| CMRW (mm) | 0.995 | 0.07 | 1.58 | 0.997 | 0.996 | 0.07 | 1.53 | 0.997 |
| Direction of the long axis of the crown (°) | 0.993 | 2.38 | 6.08 | 0.993 | 0.995 | 2.08 | 5.36 | 0.995 |
| Root axis curvature (°) | 0.996 | 1.33 | 7.28 | 0.996 | 0.995 | 1.54 | 8.59 | 0.995 |

ICC, intra-class correlation coefficient, TEM, absolute technical error of measurements, rTEM, relative technical error of measurements, R is the coefficient of reliability.
